# Patulous Eustachian tube dysfunction following rapid weight loss associated with semaglutide use: A case report

**DOI:** 10.1177/2050313X261486164

**Published:** 2026-09-02

**Authors:** James R. Burmeister, Malik Jawad, Ismail Zazay, Aaron Kobernick

**Affiliations:** 1 159878 Oakland University William Beaumont School of Medicine, Department of Foundational Studies, Rochester, MI, USA; 2 12338 University of Texas Medical Branch at Galveston, Department of Vascular Surgery, Galveston, TX, USA; 3 21818 Corewell Health William Beaumont University Hospital, Department of Internal Medicine, Royal Oak, MI, USA

**Keywords:** Patulous Eustachian tube dysfunction, weight loss, GLP-1 receptor agonist, semaglutide, autophony

## Abstract

Patulous Eustachian tube dysfunction (PETD) is an uncommon condition characterized by an abnormally patent Eustachian tube, leading to symptoms such as aural fullness and autophony. Rapid weight loss is a recognized risk factor due to loss of peritubal adipose support. With the increasing use of semaglutide and other glucagon-like peptide-1 receptor agonists for obesity management, substantial and rapid weight reduction has become more common, raising the potential for underrecognized otologic complications. A 79-year-old male presented with intermittent bilateral aural fullness and ear “popping” without associated pain, vertigo, or systemic symptoms. His history was notable for intentional weight loss of approximately 200 lb following initiation of semaglutide therapy. Otoscopic examination was unremarkable, and prior tympanometry demonstrated normal middle ear pressure. In the absence of findings suggestive of obstructive Eustachian tube dysfunction, a clinical diagnosis of patulous Eustachian tube dysfunction was made. The patient was managed conservatively with hydration, intranasal saline, and avoidance of decongestants. Rapid, extreme weight loss associated with GLP-1 receptor agonist therapy may precipitate PETD and should be considered in patients presenting with aural fullness and normal middle ear findings. Recognition of this association supports appropriate conservative management and may prevent unnecessary interventions.

## Introduction

Patulous Eustachian tube dysfunction (PETD) is a relatively uncommon otologic condition characterized by an abnormally patent Eustachian tube, resulting in symptoms such as aural fullness, autophony, and a sensation of ear “popping.” First described in the 19th century, PETD remains a diagnostic challenge due to its variable presentation and overlap with other forms of Eustachian tube dysfunction.^1^ Established etiologies include significant weight loss, pregnancy, hormonal influences, neuromuscular disorders, and structural or mucosal abnormalities of the Eustachian tube.

Among these, rapid weight loss is a well-recognized precipitating factor, thought to result from depletion of the peritubal adipose tissue that normally helps maintain closure of the Eustachian tube. While this mechanism has been described in the context of chronic illness and bariatric surgery, the relationship between acute weight loss and Eustachian tube function remains incompletely understood, with some studies suggesting variable effects on tubal physiology following surgical weight reduction.^2^

Glucagon-like peptide-1 (GLP-1) receptor agonists, such as semaglutide, have emerged as highly effective pharmacologic therapies for obesity, producing substantial and often rapid weight loss.^3^ With their increasing use, attention has turned to potential otolaryngologic manifestations associated with these agents. Recent reports suggest a possible association between GLP-1 receptor agonist–induced weight loss and the development of PETD, likely mediated by rapid loss of soft tissue bulk surrounding the Eustachian tube.^4^

Despite the growing prevalence of GLP-1 receptor agonist use, literature describing PETD in this context remains limited. A recent systematic review and database analysis identified only a small number of otologic adverse events associated with these medications and highlighted the scarcity of reported PETD cases.^5^ Given the widespread adoption of GLP-1 therapies and the potential for under-recognition of PETD, further characterization of this association is warranted.

We present a case of patulous Eustachian tube dysfunction following rapid, substantial weight loss associated with GLP-1 receptor agonist therapy, highlighting the clinical features, diagnostic considerations, and management implications of this increasingly relevant condition.

## Case Presentation

A 79-year-old male presented to an outpatient clinic with intermittent bilateral aural fullness and a sensation of “ear popping” over several weeks. The symptoms were nonpainful, occurred sporadically without a clear precipitating factor, and were not associated with hearing fluctuation, tinnitus, vertigo, nausea, or imbalance. He denied upper respiratory symptoms, chest pain, dyspnea, fevers, or chills. He did not clearly endorse autophony, though he described heightened awareness of ear fullness during symptomatic episodes. No positional triggers or relieving factors were consistently identified. The absence of overt autophony initially obscured the diagnosis, as the patient primarily emphasized the fluctuating sensation of structural pressure rather than the echoing of his own voice.

His history was notable for significant, intentional weight loss of approximately 200 lb, decreasing from 350 lb to 150 lb following initiation of semaglutide therapy (Ozempic/Wegovy), representing an aggressive average weight loss rate of approximately 16.6 lb per month. During this time, he reported increased physical activity, including regular participation in recreational sports. His past medical history was otherwise notable only for chronic bilateral sensorineural hearing loss attributed to prior military noise exposure. Prior evaluation by otolaryngology included tympanometry demonstrating normal middle ear pressure, and no definitive diagnosis had been established at that time. The onset of his mild otologic symptoms began approximately 10 months into this weight loss trajectory, as his weight loss neared its nadir.

On presentation, physical examination was unremarkable. Otoscopic examination revealed normal-appearing tympanic membranes bilaterally without evidence of effusion, retraction, or erythema. Bedside microscopic otoscopy performed during forced nasal respiration did not demonstrate obvious synchronous medial-lateral movement of the tympanic membranes at rest, and tubophony was not clinically audible during conversational examination. Given the atypical presentation, several differential diagnoses were formally considered. Obstructive Eustachian tube dysfunction was ruled out due to consistently normal Type A tympanometry curves and the lack of symptom relief with swallowing or Valsalva maneuvers. Superior semicircular canal dehiscence (SSCD) was deemed highly unlikely given the complete absence of sound- or pressure-induced vertigo (Tullio phenomenon), the absence of bone-conduction hyperacusis, and stable, symmetric pure-tone audiometry thresholds matching his long-standing baseline noise-induced sensorineural loss. Serous otitis media was confidently excluded by the absence of fluid lines, bubbles, or TM immobility. Given the patient’s history of rapid and substantial weight loss, normal middle ear pressure, and absence of findings suggestive of obstructive Eustachian tube dysfunction or inner ear pathology, a clinical diagnosis of patulous Eustachian tube dysfunction was made.

The patient was managed conservatively with counseling on adequate hydration and use of intranasal saline irrigation. He was advised to avoid decongestants, including pseudoephedrine, due to the potential to exacerbate symptoms. He was reassured regarding the typically self-limited nature of the condition and advised that symptoms may improve over time. At his 6-month follow-up, symptoms remained stable without progression or severe functional impairment, and the patient reported that the conservative measures provided adequate symptomatic buffering. Repeat tympanometry was not performed at that time given the absence of new physical exam findings or changes in middle ear status, and continued conservative management was recommended.

## Discussion

Patulous Eustachian tube dysfunction (PETD) is characterized by abnormal patency of the Eustachian tube, resulting in symptoms such as aural fullness, autophony, and perception of internal sounds.^6^ The condition arises from failure of the normal valve-like closure of the Eustachian tube, which is typically maintained by surrounding soft tissue structures, including peritubal adipose tissue. Disruption of this mechanism can lead to persistent tubal patency and the characteristic symptom profile observed in affected patients. Rapid weight loss is a well-established precipitating factor for PETD, primarily due to depletion of the peritubal fat pad that contributes to Eustachian tube closure.^7^ Prior studies evaluating patients undergoing bariatric surgery have demonstrated a significant proportion developing symptoms of Eustachian tube dysfunction following acute weight reduction, with increasing prevalence over time. These findings support the hypothesis that the rate and magnitude of weight loss play a critical role in the development of PETD. Although not all patients develop clinically significant dysfunction, the association between rapid weight loss and altered Eustachian tube mechanics is well documented. Readily compounding this, our patient’s advanced age (79 years) likely served as an important baseline risk factor. Senile atrophy of the Ostmann’s fat pad and peritubular connective tissue is a recognized physiological change in elderly populations; when combined with the severe and rapid systemic adipose depletion from GLP-1 therapy, it creates a synergistic vulnerability that precipitates acute patency.

In this context, the increasing use of semaglutide and other GLP-1 receptor agonists for obesity management introduces a novel and increasingly relevant risk factor. These agents produce substantial and sustained weight loss through appetite suppression and delayed gastric emptying, with clinical trials demonstrating significant reductions in body weight.^8^ While the safety profile of semaglutide is generally favorable, emerging evidence suggests a broader spectrum of otolaryngologic and vestibular adverse effects than previously recognized.^9,10^ The mechanism linking GLP-1 therapy to PETD is likely indirect, mediated through rapid loss of adipose tissue rather than a direct pharmacologic effect on the Eustachian tube.

The diagnosis of PETD remains primarily clinical and may be challenging due to overlap with other otologic conditions. Symptoms such as autophony may also be present in alternative pathologies, necessitating careful clinical evaluation. Objective testing, including tympanometry and more advanced modalities such as wideband tympanometry, may aid in diagnosis by demonstrating characteristic alterations in middle ear mechanics, although findings can be subtle or normal in some cases.^11^ While autophony is traditionally considered a hallmark symptom, its absence, as observed in this patient, should not rule out PETD, especially when a history of massive weight loss is present and alternative diagnoses like obstructive ETD or SSCD have been systematically excluded through negative clinical findings and normal middle ear pressures. It must be noted as a limitation of this report that advanced diagnostic tools, such as sonotubometry, wideband tympanometry during controlled respiration, or continuous nasoendoscopy of the Eustachian tube lumen, were not performed due to institutional unavailability. Nevertheless, the clinical diagnosis was securely maintained based on the classic combination of normal middle ear pressures, a history of massive rapid weight loss, and the systematic ruling out of inner ear or obstructive pathologies. This underscores the importance of thorough history-taking, particularly in identifying risk factors such as recent rapid weight loss.

Management of PETD is typically conservative, particularly in mild or early cases. First-line strategies include hydration, avoidance of medications that promote mucosal dryness, and reassurance regarding the often self-limited nature of the condition.^12^ In patients with persistent or severe symptoms, procedural interventions such as tympanic membrane mass loading or Eustachian tube augmentation techniques may be considered.^12^ Additionally, PETD has been reported as a complication following interventions aimed at treating obstructive Eustachian tube dysfunction, such as balloon dilation, further highlighting the delicate balance required for normal tubal function.^13^ This case highlights the importance of recognizing PETD as a potential consequence of rapid, medically induced weight loss. As GLP-1 receptor agonists become increasingly prevalent in the management of obesity, clinicians should maintain a high index of suspicion for PETD in patients presenting with aural symptoms and otherwise unremarkable otologic findings. Early recognition can prevent misdiagnosis and avoid unnecessary or potentially counterproductive treatments.

## Conclusion

Rapid and substantial weight loss associated with semaglutide therapy may precipitate patulous Eustachian tube dysfunction through loss of peritubal adipose support. Recognition of this association is essential to avoid misdiagnosis and inappropriate management, particularly in patients presenting with aural fullness and normal middle ear findings. Most cases can be effectively managed with conservative measures, with expectation of gradual symptomatic improvement over time. This case highlights the importance of recognizing PETD as a potential consequence of rapid, medically induced weight loss. Crucially, it introduces important management implications for prescribing clinicians. As GLP-1 receptor agonists experience explosive growth in clinical adoption, treating physicians should maintain a high index of suspicion for otologic side effects during rapid weight-loss phases. Routine clinical counseling regarding the maintenance of adequate hydration and early referral to an otolaryngologist upon the development of unexplained aural fullness can optimize conservative care. Early recognition can prevent misdiagnosis and avoid unnecessary or potentially counterproductive treatments, such as the inappropriate placement of tympanostomy tubes for suspected obstructive disease.

## Data Availability

All relevant data are included within the article.*
